# MAGE-A11 is activated through TFCP2/ZEB1 binding sites de-methylation as well as histone modification and facilitates ESCC tumor growth

**DOI:** 10.18632/oncotarget.22973

**Published:** 2017-12-05

**Authors:** Shina Liu, Fei Liu, Weina Huang, Lina Gu, Lingjiao Meng, Yingchao Ju, Yunyan Wu, Juan Li, Lihua Liu, Meixiang Sang

**Affiliations:** ^1^ Research Center, the Fourth Hospital of Hebei Medical University, Shijiazhuang 050011, P. R. China; ^2^ Animal Center, the Fourth Hospital of Hebei Medical University, Shijiazhuang 050011, P. R. China; ^3^ Tumor Research Institute, the Fourth Hospital of Hebei Medical University, Shijiazhuang 050011, P. R. China

**Keywords:** MAGE-A11, ESCC, DNA methylation, histone acetylation, histone methylation

## Abstract

Recently, we have reported that the product of Melanoma Antigens Genes (MAGE) family member MAGE-A11 is an independent poor prognostic marker for esophageal squamous cell carcinoma (ESCC). However, the reason how MAGE-A11 is activated in ESCC progression still remains unclear. In the current study, we demonstrated that DNA methylation and the subsequent histone posttranslational modifications play crucial roles in the regulation of MAGE-A11 in ESCC progression. We found that the methylation rate of TFCP2/ZEB1 binding site on MAGE-A11 promoter in ESCC tissues and cells is higher than the normal esophageal epithelial tissues and cells. Transcription factors TFCP2 and ZEB1 directly bind MAGE-A11 promoter and regulate the endogenous MAGE-A11 expression in a methylation-dependent manner in ESCC cells. Following MAGE-A11 promoter methylation, the methyl-CpG-binding protein MeCP2 was found to bind the methylated MAGE-A11 promoter to mediate histone deactylation by recruiting HDAC1 and HDAC2. Simultaneously, histone inactivation marks including H3K27me3 as well as H3K9me3 were increased, whereas histone activation mark H3K4me3 was decreased. HDAC inhibitor Trichostatin A (TSA) increased DNA methylase inhibitor Decitabine (DAC)-induced MAGE-A11 expression. siRNA-mediated knockdown of histone methltransferase EZH2 or DZNep (a EZH2 inhibitor) treatment increased DAC-induced MAGE-A11 expression. Our results indicate that MAGE-A11 is activated through DNA demethylation, histone acetylation and histone methylation in ESCC, and its activation promotes ESCC tumor growth.

## INTRODUCTION

The Melanoma Antigens Genes (MAGE) proteins are a group of highly conserved family members that contain a common MAGE homology domain (MHD) [1]. Type I MAGEs are relevant cancer-testis antigens (CTAs), and originally considered as attractive targets for cancer immunotherapy due to their typically high expression in tumor tissues but restricted expression in normal adult tissues [2–4]. In addition to their significance as cancer immunotherapeutic targets, MAGE gene products may also contribute to cancer progression as oncoproteins [5, 6]. In particular, MAGE proteins bind directly to RING family of ubiquitin E3 ligase, thus regulating the E3 ubiquitin ligase activity and triggering the ubiquitination and degradation of multiple tumor suppressors, such as p53 and AMPKα1, promoting tumorigenesis and aggressive tumor growth [7–11]. In addition, MAGE proteins also interact with transcription factors and function as co-regulators in cancer progression [12–15].

MAGE-A11, one of MAGE family members, was found to form complex with androgen receptor (AR) and increase the AR transcriptional activity [16, 17]. The increased expression of MAGE-A11 facilitates prostate cancer progression by enhancing AR-dependent tumor growth [18]. In addition, MAGE-A11 also functions as a transcriptional coactivator of progesterone receptor (PR), and its capacity is in part mediated through interaction with p300 histone acetyltransferase [19, 20]. MAGE-A11 expression could also serve as a cancer prognostic marker, based on our previous data showing that MAGE-A11 was correlated with tumor progression and reduced survival [21, 22]. We previously reported that MAGE-A11 is an independent poor prognostic marker for esophageal squamous cell carcinoma (ESCC) patients, and directly increase the invasion and proliferation of ESCC cells [22]. However, the regulation mechanism of MAGE-A11 in ESCC progression is still unclear.

Although believed to be activated as a result of global DNA demethylation, the epigenetic mechanisms coordinates de-repression of *MAGE* genes during cancer progression have not been fully elucidated [23–25]. In addition, DNA methylation is intertwined with the posttranslational modifications of histone [26, 27]. Studies aimed to discern the relationship among these interdependent epigenetic mechanisms of *MAGE-A11* gene are limited.

The present study was undertaken to comprehensively examine mechanisms regulating MAGE-A11 expression in ESCC cells. We discovered that transcription factors TFCP2 and ZEB1 directly bind MAGE-A11 promoter and regulate MAGE-A11 expression in a methylation-dependent manner in ESCC. The subsequent post-translational modifications of histone including histone acetylation and methylation followed DNA methylation are also involved in the activation of MAGE-A11. HDAC inhibitor Trichostatin A (TSA) and histone methltransferase EZH2 inhibitor DZNep increase the DNA methylase inhibitor 5-aza-2’ deoxycytidine (Decitabine, DAC)-induced MAGE-A11 expression. Our study provided insight into the role of MAGE-A11 in ESCC, and strengthens the possible clinical potential from postoperative vaccine targeting MAGE-A11 combined with epigenetic agents in ESCC.

## RESULTS

### MAGE-A11 is associated with poor prognosis of ESCC and increases the ESCC xenograft tumor growth

**Figure 1:**
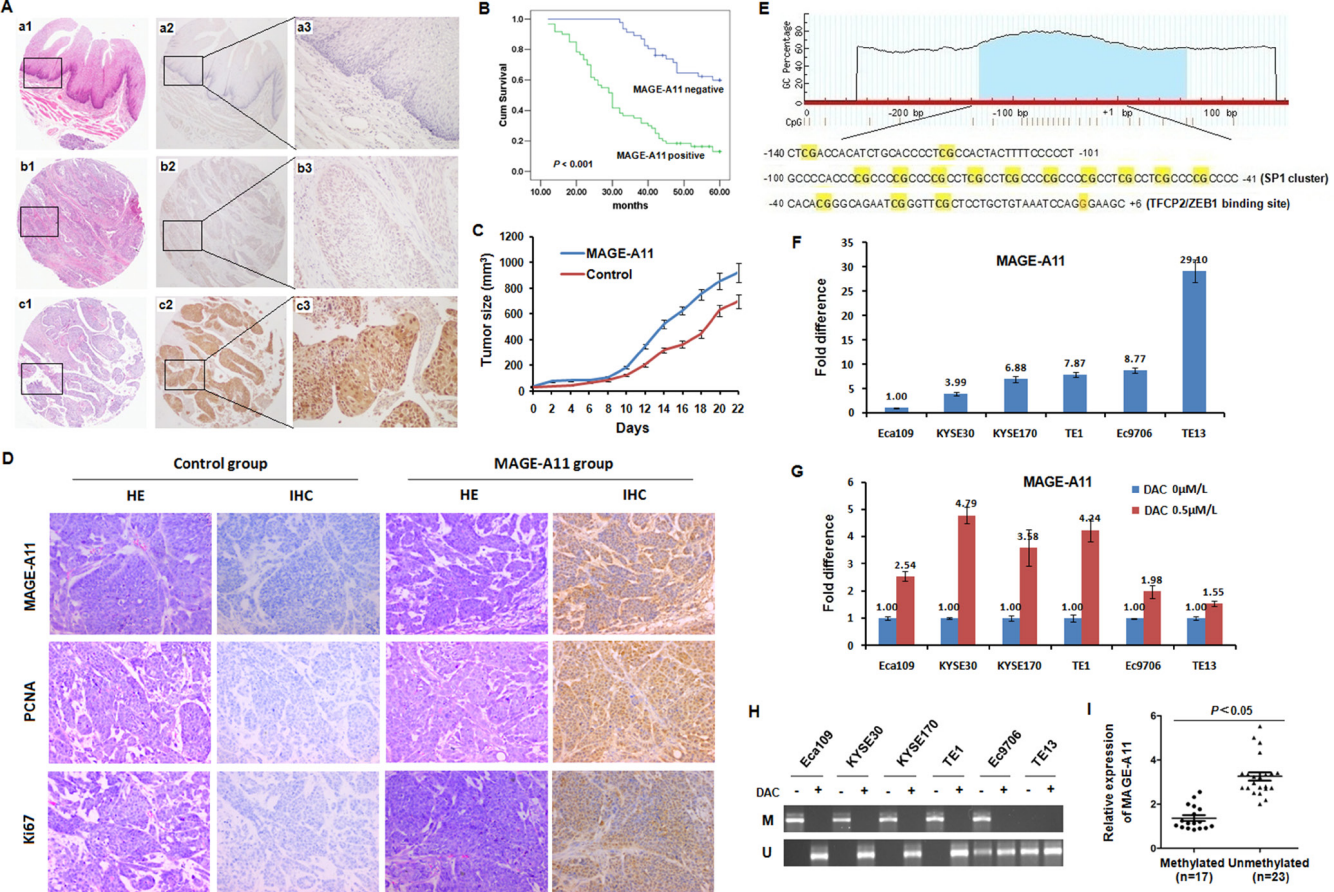
MAGE-A11 promotes ESCC growth and its expression is associated with promoter methylation (**A**) HE staining and IHC staining of MAGE-A11 in TMA. (a1) HE staining (×10), (a2) MAGE-A11 IHC staining (×10), and (a3) MAGE-A11 IHC staining (×20), of pericarcinoma esophageal tissue; (b1) HE staining (×10), (b2) Negative IHC staining for MAGE-A11 (×10), and (b3) Negative IHC staining for MAGE-A11 (×20), of ESCC tissues; (c1) HE staining (×10), (c2) Positive IHC staining for MAGE-A11 (×10), and (c3) Positive IHC staining for MAGE-A11 (×20), of ESCC tissue. (**B**) MAGE-A11 expression is associated with poor survival of ESCC patients. (**C**) Tumor size and tumor growth of Eca109 mice xenograft carrying MAGE-A11. (**D**) IHC showed the expression of Ki67, PCNA and MAGE-A11 in Eca109 mice xenograft tissues. (**E**) One around 200 bp of CpG island was shown on the promoter and Exon1 of MAGE-A11. The region from -140 to +1 on MAGE-A11 promoter contains fifteen CG sites and several transcription factors binding sites including SP1 cluster (10 embedded CpG sites) and a big TFCP2 and ZEB1 binding site which covers three CpG sites following the SP1 cluster. (**F**) Expression of MAGE-A11 in ESCC cell lines detected by qRT-PCR. (**G**) ESCC cells were treated with 0.5 μM/L of DAC for 48 h, and the expression of MAGE-A11 detected by qRT-PCR. (**H**) MSP method showed the methylation status of ESCC cell lines before and after 0.5 μM/L of DAC treatment for 48 h. (**I**) The relative expression of MAGE-A11 in 40 selected cases of ESCC tissues divided into methylated and unmethylated group based on MSP.

Previously, we have detected MAGE-A11 expression in ESCC tissues by IHC staining and found MAGE-A11 is an independent poor prognostic marker for ESCC. To eliminate the staining difference at different time, we confirmed the expression pattern and clinical relevance of MAGE-A11 in ESCC patients by using TMA-based immunohistochemistry in 106 pairs of ESCC tissues and the corresponding adjacent normal esophageal epithelial tissues. Our present study showed that MAGE-A11 is not expressed in the normal esophageal epithelial tissues, but expressed in 56.6% of ESCC tissues (Figure 1A; Supplementary Table 1). Furthermore, we found that MAGE-A11 expression was positively associated with tumor invasion, lymph node metastasis, distant metastasis or recurrence, TNM stage, histological grade of ESCC patients (Supplementary Table 2). In addition, ESCC patients with positive expression of MAGE-A11 have significantly reduced 5-year overall survival (*P* < 0.001; Figure 1B). A multivariate Cox regression analysis showed that MAGE-A11 expression is an independent poor prognostic factor for ESCC (*P* < 0.001; Supplementary Table 3).

To extend our observation in clinical specimens, we investigated whether MAGE-A11 could regulate esophageal tumor growth in animal experiment. The esophageal cancer Eca109 cells bearing enforced MAGE-A11 expression and their corresponding control cells were subcutaneously injected into nude mice. As shown in Figure 1C and Supplementary Figure 1, the xenografts formed by MAGE-A11 overexpression cells revealed increased cell growth than the control tumors. In addition, MAGE-A11 overexpression xenografts showed higher expression of cell proliferation markers including Ki67 and PCNA, than the control tumors (Figure 1D). These data suggested that MAGE-A11 is a poor prognostic marker for ESCC and increases the tumor growth and cell proliferation of ESCC.

### MAGE-A11 expression is associated with promoter hypomethylation in ESCC cells

Although the activation mechanism of MAGE-A11 in ESCC is still unclear, the promoter methylation seems to play a crucial role in the activation of MAGE family in cancer progression. Commonly, CpG islands are frequently silenced by promoter or exon1 methylation as an alternative epigenetic mechanism to suppress gene expression and inactivate gene functions. Therefore, we used MethPrimer program and the CpG Island Search to determine whether MAGE-A11 gene contains CpG islands [28, 29]. As shown in Figure 1E, one around 200bp of CpG island was found on the promoter and exon1 region of MAGE-A11. Therefore, we considered promoter hypomethylation might be a regulation mechanism of MAGE-A11 in ESCC. We next examined the expression of MAGE-A11 in six ESCC cell lines by qRT-PCR. As shown in Figure 1F, MAGE-A11 mRNA is expressed in ESCC cells at different level. Among these cells, Eca109 and KYSE30 showed relatively low expression of MAGE-A11, while TE13 showed relatively high expression of MAGE-A11. After treated with the demethylation agent DAC, MAGE-A11 mRNA was induced at different level in all ESCC cells. In MAGE-A11 low-expressed cells, MAGE-A11 was induced by DAC treatment, whereas it was only slightly induced in MAGE-A11 high-expressed cells (Figure 1G). Then, we performed MSP analysis to determine the methylation status of the CpG island on MAGE-A11 promoter in ESCC cells. As shown in Figure 1H, this region was highly methylated in all these cell lines except for TE13 cells. After DAC treatment, this region were demethylated (Figure 1H). We then selected 40 cases of ESCC tissues to detect the methylation status of this region by using MSP, and examined MAGE-A11 expression by using RT-qPCR. As shown in Figure 1I, the expression level of MAGE-A11 was higher in the unmethylated group than the methylated group. Taken together, these date suggested that DNA methylation plays a crucial role in the regulation of MAGE-A11 expression in ESCC cells.

### MAGE-A11 expression is highly associated with hypomethylation of the region from -140 to +1 on MAGE-A11 promoter in ESCC cells

**Figure 2:**
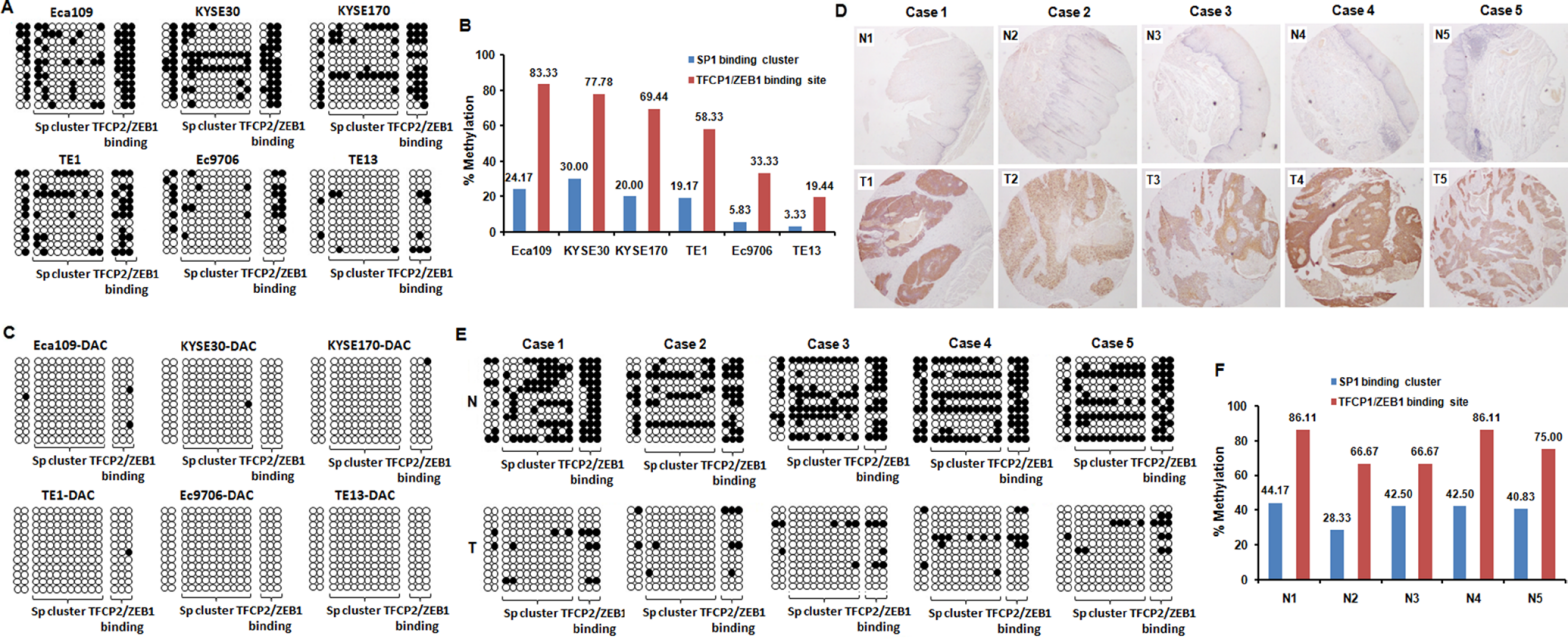
MAGE-A11 expression is highly associated with hypomethylation of the region from -140 to +1 on MAGE-A11 promoter in ESCC cells (**A**) Bisulfite clonal sequencing showed the methylation status of the fifteen CG sites from -140 to +1 on MAGE-A11 promoter in ESCC cell lines. (**B**) The methylation rate of the fifteen CG sites from -140 to +1 on MAGE-A11 promoter in ESCC cell lines. (**C**) Bisulfite clonal sequencing showed the methylation status of the fifteen CG sites from -140 to +1 on MAGE-A11 promoter after 0.5 μM/L of DAC treatment for 48 h in ESCC cell lines. (**D**) The IHC staining showed the selected 5-paired MAGE-A11 positive ESCC tissues and MAGE-A11 negative pericarcinoma esophageal tissues for bisulfite clonal sequencing. (**E**) Bisulfite clonal sequencing showed the methylation status of the fifteen CG sites from -140 to +1 on MAGE-A11 promoter in the selected 5-paired ESCC tissues and pericarcinoma esophageal tissues. (**F**) The methylation rate of the fifteen CG sites from -140 to +1 on MAGE-A11 promoter in the selected 5-paired ESCC tissues and pericarcinoma esophageal tissues.

For further analyze which CpG sites have a key role in the regulation of MAGE-A11 expression, the BSP method was performed in all these six ESCC cells to detect the methylation status of the fifteen CG sites from -140 to +1 on MAGE-A11 promoter. As shown in Figure 2A, this CpG island was moderately to highly methylated in TE1, KYSE170, KYSE30, Eca109 cells which had relatively low level of MAGE-A11 expression, while this region was hypomethylated in Ec9706 and TE13 cells expressing high level of MAGE-A11. For further analysis which transcription factors regulate MAGE-A11 activity in a methylation-dependent manner, we then analyzed the transcription factor binding sites of this 200bp of CpG island covering MAGE-A11 promoter and exon1 through JASPAR online database [30]. This region contains a SP1 cluster (10 embedded CpG sites) which has been reported to active MAGE-A11 expression in prostate cancer cells in a methylation-dependent manner [31]. There also exists a TFCP2 and ZEB1 binding site which covers three CpG sites following the SP1 cluster (Figure 1E). Our sequencing results showed that the methylation rate of TFCP2/ZEB1 binding region was higher than the SP1 binding cluster in all these ESCC cells (Figure 2B). After 0.5 μM/L of DAC treatment, these CpG sites were dramatically demethylated (Figure 2C). In addition, we also detected the hypermethylation of these CpG sites in normal esophageal epithelial HEEC cells, and DAC treatment induced the demethylation of MAGE-A11 promoter (Supplementary Figure 2). Our results suggested that the TFCP2/ZEB1 binding region had a higher methylation frequency than SP1 binding cluster in ESCC cells.

For further confirming this notion, we selected five-paired MAGE-A11 highly expressed ESCC tissues and the corresponding adjacent normal esophageal epithelial tissues, and collected the tumor cells and normal epithelial cells by using laser-capture microdissection (Figure 2D). Bisulfite clonal sequencing results showed that these fifteen CG sites from -140 to +1 on MAGE-A11 promoter was highly methylated in normal esophageal epithelial tissues, but hypomethylated in ESCC cells (Figure 2E). The methylation rate of TFCP2/ZEB1 binding region was higher than the SP1 binding cluster in normal epithelial cells (Figure 2F). Taken together, our results indicated that MAGE-A11 expression in ESCC cells is highly associated with DNA hypermethylation of its promoter region, especially the TFCP2/ZEB1 binding region.

### Transcription factors TFCP2 and ZEB1 directly bind MAGE-A11 promoter in a methylation-dependent manner in ESCC cells

**Figure 3:**
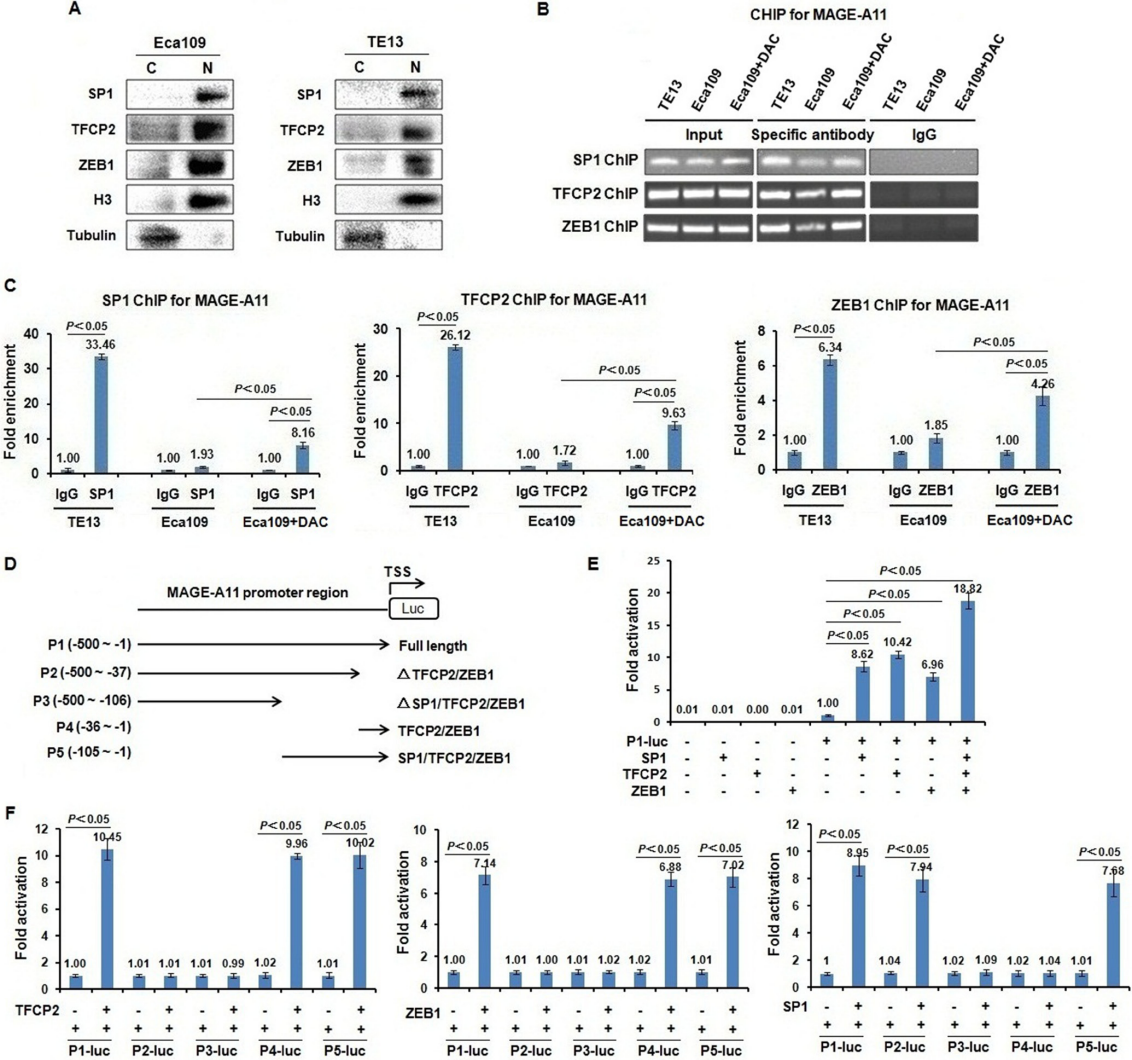
Transcription factors TFCP2 and ZEB1 directly bind MAGE-A11 promoter and regulate the transcription-mediated luciferase activity of MAGE-A11 in a methylation-dependent manner in ESCC cells (**A**) The sub-cellular localization experiments showed that TFCP2, ZEB1 and SP1 were detectable largely in cell nucleus. (**B**) and (**C**) ChIP results showed the binding of SP1, TFCP2 and ZEB1 on the promoter of MAGE-A11 in TE13 and Eca109 cells before and after 0.5 μM/L of DAC treatment for 48 h. (**D**) The construction of luciferase reporters carrying deletion mutants of MAGE-A11 promoter. (**E**) The luciferase reporter experiment showed that in the presence of full length of MAGE-A11 promoter (–500∼–1), the luciferase activity driven by MAGE-A11 promoter was induced by the transcription factors including TFCP2, ZEB1 and SP1, respectively. (**F**) The luciferase activity driven by deletion mutants of MAGE-A11 promoter.

To confirm whether MAGE-A11 is a direct target of TFCP2 and ZEB1, we detected the localization of these transcription factors, and then carried out ChIP assay in Eca109 and TE13 cells to verify whether TFCP2 and ZEB1 directly bind to MAGE-A11 promoter in a methylation-dependent manner. As shown in Figure 3A and Supplementary Figure 3, TFCP2, ZEB1 and SP1 were detectable largely in cell nucleus. Transcription factor SP1 has been reported to active MAGE-A11 expression in prostate cancer cells in a methylation-dependent manner. Therefore, we performed the experiment of SP1 ChIP for MAGE-A11 promoter as the positive control. ChIP results showed that the specific binding of SP1 to MAGE-A11 promoter was increased as compared with IgG control in TE13 cells which showed high expression of MAGE-A11 and hypomethylation of MAGE-A11 promoter (33.46 of fold enrichment) (Figure 3B and 3C). In Eca109 cells which showed low expression of MAGE-A11 and hypermethylation of MAGE-A11 promoter, the specific binding of SP1 to MAGE-A11 promoter was only slightly increased as compared with IgG control (1.93 of fold enrichment), whereas after DAC treatment, the specific binding of SP1 to MAGE-A11 promoter was increased (8.16 of fold enrichment). Similarly, the specific binding of TFCP2 to MAGE-A11 promoter was increased as compared with IgG control in TE13 cells (26.21 of fold enrichment) (Figure 3B and 3C). In Eca109 cells, the specific binding of TFCP2 to MAGE-A11 promoter was only slightly increased as compared with IgG control (1.72 of fold enrichment), whereas DAC treatment could increase the specific binding of TFCP2 to MAGE-A11 promoter in Eca109 cells (9.63 of fold enrichment), suggesting that TFCP2 could directly bind to MAGE-A11 promoter in a methylation-dependent manner in ESCC cells. Similar results were found in the experiment of ZEB1 ChIP for MAGE-A11 promoter (Figure 3B and 3C). These date suggested that, similar with SP1, TFCP2 and ZEB1 directly bind MAGE-A11 promoter in a methylation-dependent manner in ESCC cells.

### TFCP2 and ZEB1 directly regulate the transcription activity of MAGE-A11

To further confirm whether the binding of TFCP2 and ZEB1 to MAGE-A11 promoter could regulate the transcription activity of MAGE-A11, we constructed the luciferase reporters carrying deletion mutants of MAGE-A11 promoter (Figure 3D), and performed the *in vitro* luciferase reporter assay. As shown in Figure 3E, in the presence of full length of MAGE-A11 promoter (-500∼-1, P1) that covers -140∼-1 region, the luciferase activity driven by MAGE-A11 promoter was induced by the transcription factors including TFCP2, ZEB1 and SP1, respectively, suggesting that these transcription factors could directly regulate MAGE-A11 promoter-mediated luciferase activity. This result was further confirmed by the luciferase reporter assay carrying deletion mutants of MAGE-A11 promoter (Figure 3F).

### TFCP2 and ZEB1 regulate MAGE-A11 transcription in a methylation-dependent manner in ESCC cells

**Figure 4:**
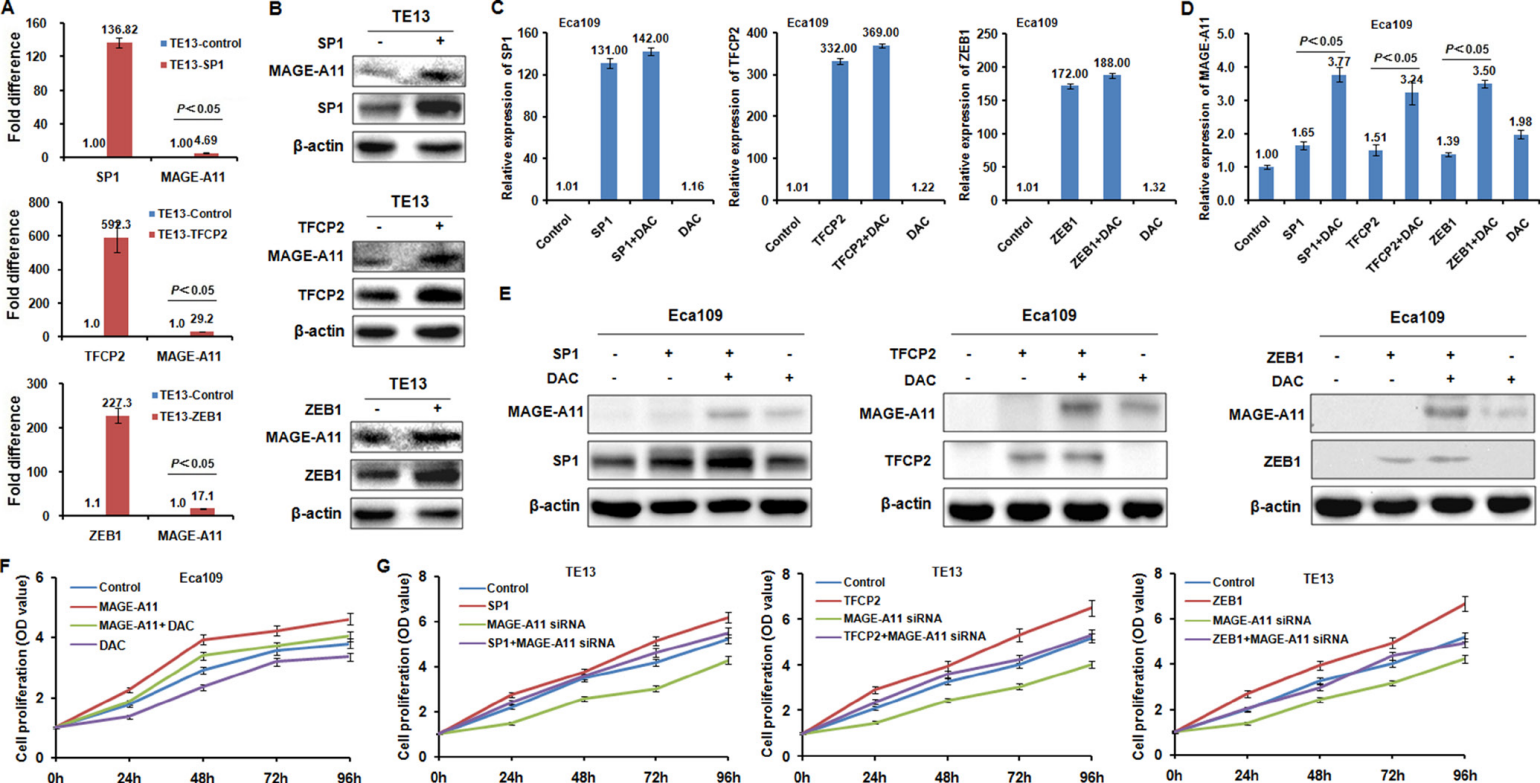
TFCP2 and ZEB1 regulate MAGE-A11 transcription in a methylation-dependent manner in ESCC cells (**A**) qRT-PCR results showed the expression of MAGE-A11 before and after TFCP2, ZEB1 and SP1 overexpression at the mRNA level in TE13 cells. (**B**) Western blot results showed the expression of MAGE-A11 before and after TFCP2, ZEB1 and SP1 overexpression at the protein level in TE13 cells. (**C**) and (**D**) showed the expression of MAGE-A11 after the treatment of TFCP2, ZEB1 and SP1 overexpression as well as 0.5 μM/L of DAC treatment for 48h by using qRT-PCR and western blot. (**E**) Western blot experiments confirmed the results in Figure4 C and 4D at protein level. (**F**) Cell proliferation assay showed the cell proliferation after MAGE-A11 overexpression and 0.5 μM/L of DAC treatment in Eca109 cells. (**G**) Cell proliferation assay showed the cell proliferation after MAGE-A11 siRNA knockdown and overexpression of TFCP2, ZEB1 and SP1 in TE13 cells.

To address whether these transcription factors regulate MAGE-A11 transcription activity in a methylation-dependent manner in ESCC cells, we transfected TFCP2, ZEB1 and SP1 expression plasmid in TE13 cells which carried hypomethylation of MAGE-A11 promoter. Our results showed that enforced expression of TFCP2, ZEB1 and SP1 induced MAGE-A11 expression at the mRNA level and protein level, respectively (Figure 4A and 4B). However, in Eca109 cells which carried hypermethylation of MAGE-A11 promoter, MAGE-A11 was not or only slightly induced by TFCP2, ZEB1 and SP1 at the mRNA level and protein level, respectively (Figure 4C, 4D and 4E). However, DAC treatment could increase the transcription factors-induced MAGE-A11 expression (Figure 4D and 4E). These date suggested that transcription factors TFCP2 and ZEB1 regulate MAGE-A11 expression in a methylation-dependent manner in ESCC cells.

In addition, MTT results showed that overexpression of MAGE-A11 increased cell proliferation, whereas siRNA-mediated knockdown of MAGE-A11 decreased cell proliferation of ESCC cells (Figure 4F and 4G). Although DAC treatment increased the transcription factors-induced MAGE-A11 expression of Eca109 cells (Figure 4D and 4E), it decreased the cell proliferation of Eca109 cells, which may be due to the demethylation effects of DAC on other tumor suppressors that are always methylated in tumor cells [32, 33]. In TE13 cells, the transcription factors including SP1, TFCP2 and ZEB1 could increase the cell proliferation. However, after MAGE-A11 siRNA treatment, these transcription factors failed to increase the cell proliferation of TE13 cells. Taken together, our results suggested that these TECP2 and ZEB1 regulate ESCC cells proliferation at least partly through inducing MAGE-A11 transcription.

### MAGE-A11 is regulated through the combination of DNA methylation and histone acetylation in ESCC cells

**Figure 5:**
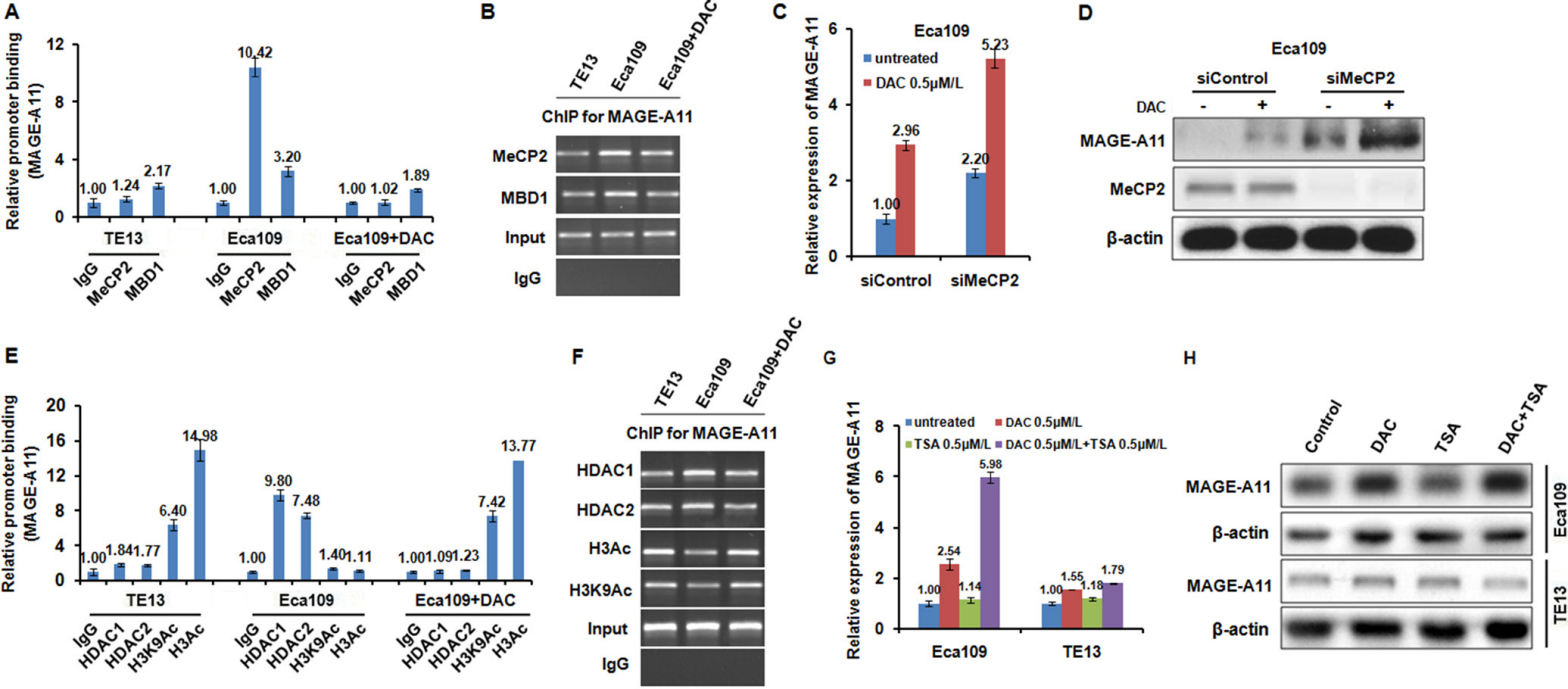
MAGE-A11 is regulated through the combination of DNA methylation and histone acetylation in ESCC cells (**A**) and (**B**) ChIP results showed the binding of MeCP2 and MBD1 on the promoter of MAGE-A11 in TE13 and Eca109 cells before and after 1 μM/L of DAC treatment for 48 h. (**C**) and (**D**) showed the expression of MAGE-A11 after combination treatment of MeCP2 siRNA and 0.5 μM/L of DAC treatment for 48h, detected by qRT-PCR and Western blot. (**E**) and (**F**) ChIP results showed the binding of HDAC1, HDAC2, H3K9Ac, H3Ac on the promoter of MAGE-A11 in TE13 and Eca109 cells before and after 0.5 μM/L of DAC treatment for 48 h. (**G**) and (**H**) showed the expression of MAGE-A11 after treatment of 0.5 μM/L of TSA and 0.5 μM/L of DAC treatment for 48 h, detected by qRT-PCR and Western blot.

Following methylation, methyl-CpG-binding proteins (MBPs), such as MBD1, MBD2, MeCP2 are commonly recruited to CpG sites, and repress transcription by recruiting Sin3A, which interacts with histone deacetylases (HDACs), and form a corepressor complex [34]. To investigate which MBPs were recruited to the methylated CpG sites of MAGE-A11 promoter, we performed ChIP analysis using antibodies targeting two MBPs including MBD1 and MeCP2. As shown in Figure 5A and 5B, MeCP2 binds to the hypermethylated MAGE-A11 promoter in Eca109 cells, but not bind to the hypomethylated MAGE-A11 promoter in TE13 cells, and DAC treatment results in the decreased MeCP2 binding to the promoter of MAGE-A11 in Eca109 cells. However, MBD1 binds to MAGE-A11 promoter regardless of the methylation status. In addition, siRNA-mediated knockdown of MeCP2 increased the DAC-induced MAGE-A11 expression at mRNA level and protein level (Figure 5C and 5D).

**Figure 6:**
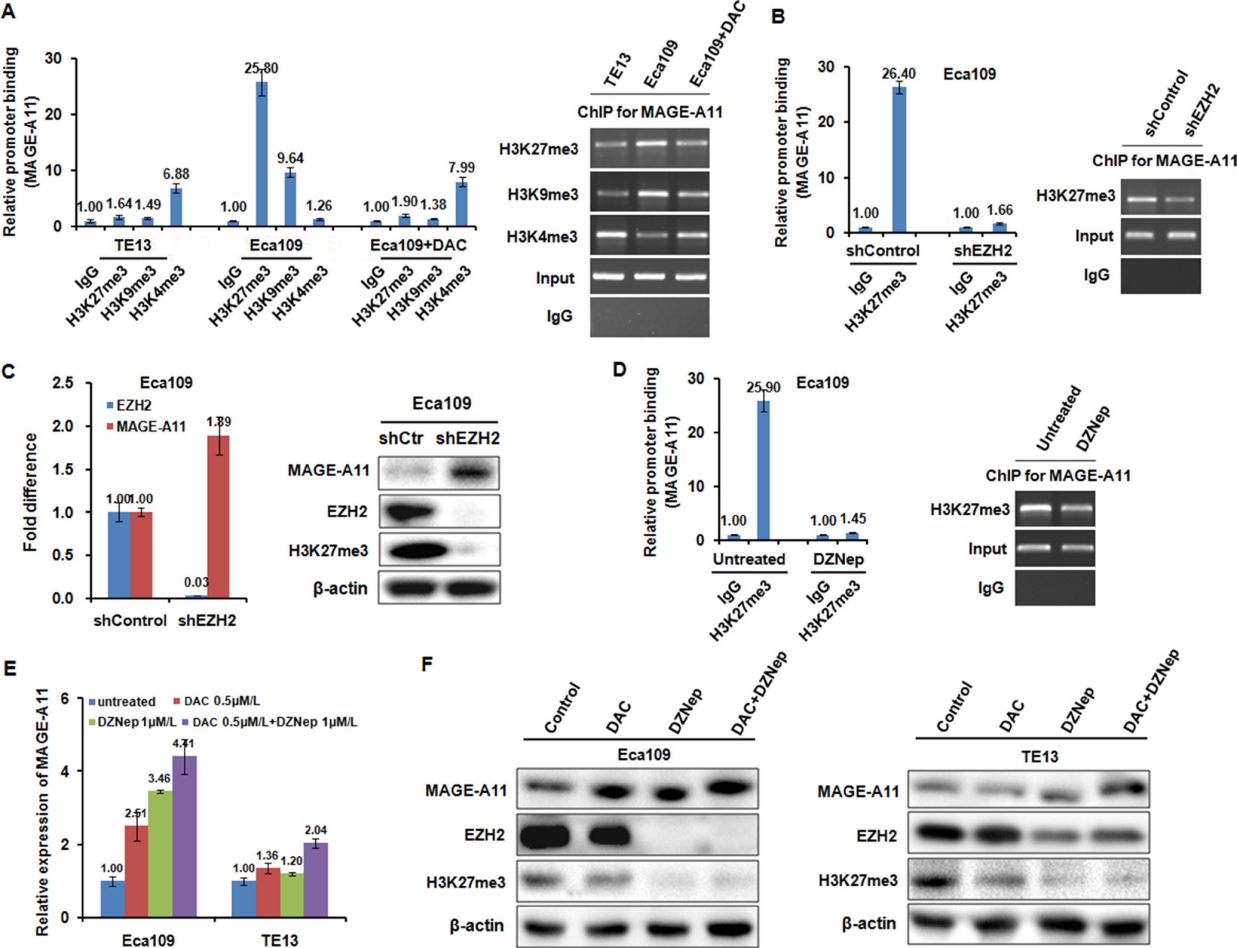
Histone methylation is participated into the regulation of MAGE-A11 transcription in ESCC cells (**A**) ChIP results showed the binding of H3K27me3, H3K9me3, and H3K4me3 on the promoter of MAGE-A11 in TE13 and Eca109 cells before and after 0.5 μM/L of DAC treatment for 48 h. (**B**) ChIP results showed the binding of H3K27me3 on the promoter of MAGE-A11 in Eca109 cells after treatment with EZH2 siRNA. (**C**) qRT-PCR and western blot showed the expression of MAGE-A11 after treatment with EZH2 siRNA. (**D**) ChIP results showed the binding of H3K27me3 on the promoter of MAGE-A11 in Eca109 cells after 1 μM/L of DZNep treatment for 48 h. (**E**) and (**F**) qRT-PCR and western blot showed the expression of MAGE-A11 after treatment of 1 μM/L of DZNep and 0.5 μM/L of DAC treatment for 48 h.

Subsequently, to investigate whether HDACs, known to be repressors of transcription, were related the MAGE-A11 promoter methylation, ChIP analysis was performed using antibodies targeting HDAC1, HDAC2, H3K9Ac, and H3Ac in Eca109 and TE13 cells. As shown in Figure 5E and 5F, HDAC1 and HDAC2 bind to the hypermethylated MAGE-A11 promoter in Eca109 cells, but not bind to the hypomethylated MAGE-A11 promoter in TE 13 cells. DAC-induced demethylation of MAGE-A11 promoter blocks binding of HDAC1 and HDAC2 in Eca109 cells. Simultaneously, H3K9Ac and H3Ac bind to the hypomethylated MAGE-A11 promoter in TE 13 cells, but not bind to the hypermethylated MAGE-A11 promoter in Eca109 cells. DAC-induced demethylation of MAGE-A11 promoter increased the binding of H3K9Ac and H3Ac in Eca109 cells. In addition, in MAGE-A11 hypermethylated Eca109 cells, the HDAC inhibitor TSA treatment increased the expression of MAGE-A11 when combined with DAC, although it could not enhance MAGE-A11 expression alone (Figure 5G and 5H). However, in MAGE-A11 hypomethylated TE13 cells, TSA treatment could not affect the expression of MAGE-A11 individually or in combination (Figure 6G and 6H). These data suggested that MAGE-A11 is regulated through the combination of DNA methylation and histone acetylation in ESCC cells.

### Histone methylation is involved in the regulation of MAGE-A11 transcription in ESCC cells

**Figure 7:**
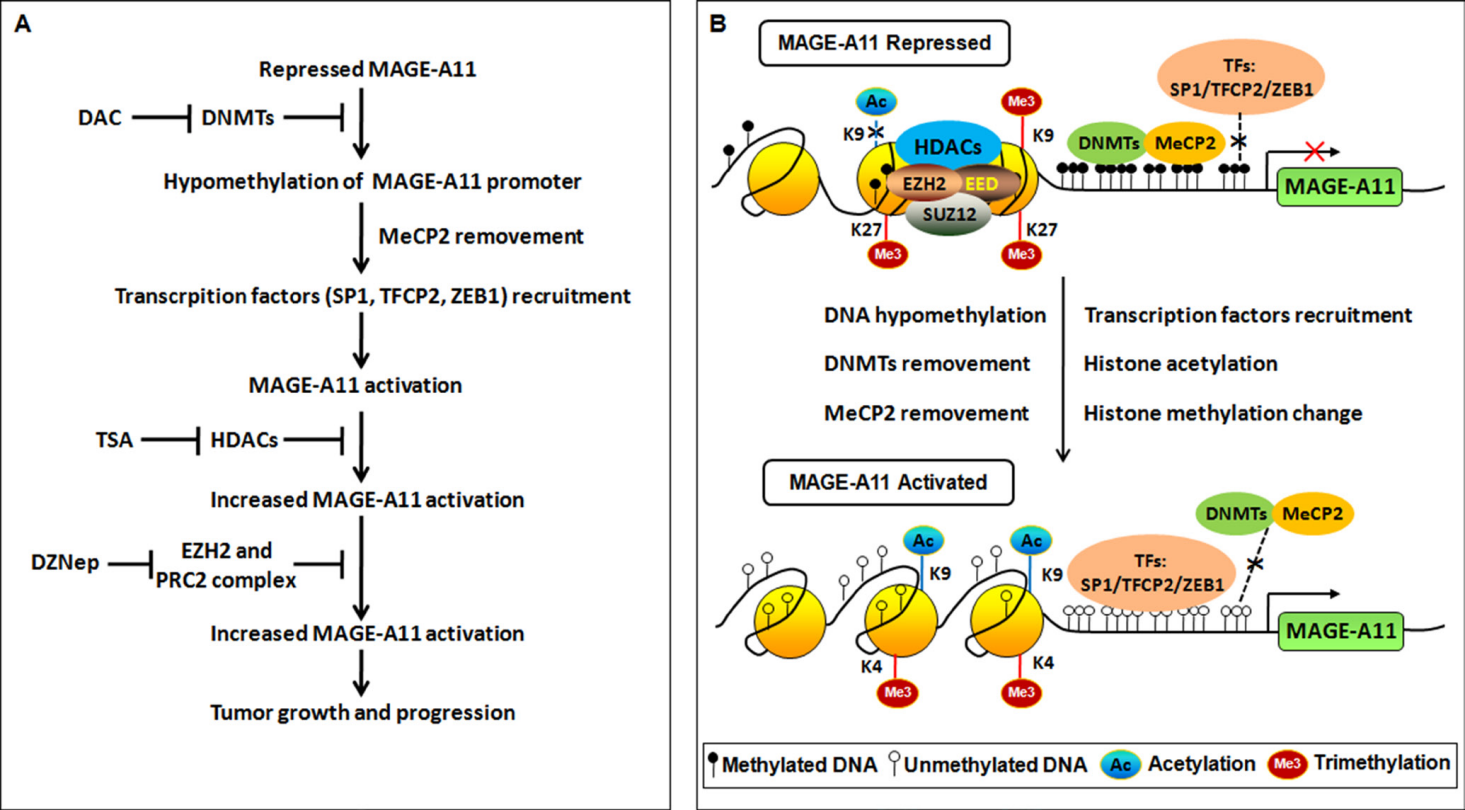
Regulation of MAGE-A11 gene in ESCC (**A**) Flowchart of epigenetic events and the associated agents involved in MAGE-A11 activation in ESCC. (**B**) Schematic of epigenetic events involved in MAGE-A11 activation in ESCC.

DNA methylation is intertwined with the post translational modification of histone including histone acethylation, and histone methylation [26, 27]. Moreover, MeCP2 cooperates in maintenance of a repressive chromatin state by acting as a link between DNA and histone methylation [35]. Therefore, we carried out ChIP analysis using antibodies targeting H3K27me3, H3K9me3, H3K4me3 in Eca109 and TE13 cells. According to the results of Figure 6A, MAGE-A11 promoter in Eca109 cells exhibited increased occupancy of inactivation marks such as H3K27me3 as well as H3K9me3, and decreased occupancy of activation mark H3K4me3. In contrast, MAGE-A11 promoter in TE13 cells exhibited decreased occupancy of H3K27me3 as well as H3K9me3, and increased occupancy of activation mark H3K4me3. After treatment with DAC, the occupancy of H3K27me3 as well as H3K9me3 on MAGE-A11 promoter in Eca109 cells was decreased, whereas the occupancy of H3K4me3 was increased. When we knockdown EZH2 which is a component of polycomb repressor complex (PRC)-2 and mediate trimethylation of H3K27, the occupancy of H3K27me3 on MAGE-A11 promoter was decreased (Figure 6B), and the expression of MAGE-A11 was increased (Figure 6C). DZNep, a pharmacologic inhibitor of EZH2, recapitulated the effects of EZH2 knockdown (Figure 6D). In addition, in MAGE-A11 hypermethylated Eca109 cells, DZNep treatment increased the expression of MAGE-A11 when combined with DAC as compared with the DZNep treatment alone (Figure 6E and 6F). In MAGE-A11 hypomethylated TE13 cells, DZNep treatment could not affect the expression of MAGE-A11 individually or in combination (Figure 6E and 6F), suggesting that histone methylation is a subsequent event after DNA methylation. Taken together, our present data demonstrated that DNA demethylation plays a primary role, and the subsequent histone modifications including histone acetylation and histone methylation changes play accessory roles in MAGE-A11 activation in ESCC progression (Figure 7). These epigenetic changes promote the re-expression of MAGE-A11 and facilitate the cell proliferation and tumor growth of ESCC.

## DISCUSSION

In the present study, we demonstrated that MAGE-A11 increased the tumor growth and cell proliferation of ESCC *in vivo*. In addition, we elucidated the epigenetic mechanisms regulating MAGE-A11 expression in ESCC cells. MAGE-A11 activation in cancer progression occurs in conjuction with the activation of other CTA genes. DNA methylation is the major epigenetic mechanism silencing CTA genes in normal somatic cells, and CTA genes expression can be induced in cancer cells by DNA demethylating agents or knockdown of (DNA methyltransferases) DNMTs [24]. As suggested by James et al., CpG island of MAGE-A11 promoter is hypermethylated in benign prostate intraepithelial neoplasia, but hypomethylated in prostate cancer, especially at the transcription start site (TSS)-resident CpG sites [31, 36]. These TSS-resident CpG sites include the SP1 transcription binding sites, and contribute to MAGE-A11 promoter activity and endogenous gene expression of MAGE-A11. In addition, in prostate cancer, DNA methylation regulates nucleosome occupancy specifically at the -1 positioned necleosome of MAGE-A11, therefore strongly repressing MAGE-A11 promoter activity. In the present study, we have found that beyond the SP1 binding cluster, TFCP1/ZEB1 binding site which covers three CpG sites following the SP1 cluster seems to have a higher methylation rate in normal esophageal epithelial tissues and some MAGE-A11 low-expressed ESCC cells than the SP1 cluster. MAGE-A11 promoter activity and endogenous expression could be directly induced by the enforced expression of transcription factors TFCP2 and ZEB1 in a methylation-dependent manner. The transcription factor TFCP2, also known as LSF, is involved in many biological events, including cell cycle, cell growth and development, through acting as transcription activator or repressor [37, 38]. In cacer-related studies, TFCP2 was reported to act as an oncogene in hepatocellular carcinoma, pancreatic cancer, and colorectal cancer [39, 40, 41]. ZEB1 is a transcription factor that promotes tumor invasion and metastasis by inducing epithelial-mesenchymal transition (EMT) in carcinoma cells. Through zinc finger clusters, ZEB1 can bind to specific DNA sequences. By recruiting co-suppressors or co-activators, ZEB1 can either downregulate or upregulate the expression of its target genes [42, 43]. For instance, ZEB1 directly binds to the promoter of *CDH1 and TAp73,* leading to repression of *CDH1* and TAp73 transcription [43, 44]. On the other hand, by recruiting p300-P/CAF and Smad proteins, ZEB1 can activate the transcription of TGF-β-responsive genes and promote osteoblastic differentiation [42, 45].

Although hypermethylation of CpG-rich MAGE gene promoters plays a crucial role in the silencing of MAGE genes, up-regulation of MAGE gene could not be always observed although tumor cells were treated by the DNA methylase inhibitors [46]. Histone deacetylases inhibitor TSA was able to up-regulate DNA methylase inhibitors-induced MAGE gene transcription, although treatment of several tumor cells with TSA alone had only small influence on MAGE gene expression [47]. Following methylation, some MBPs are commonly recruited to CpG sites, and repress transcription by recruiting HDACs [34]. According to our present results, MeCP2, but not MBD1 binds to the hypermethylated MAGE-A11 promoter in Eca109 cells, but not to the hypomethylated MAGE-A11 promoter in TE 13 cells. The treatment of demethylating agents causes hypomethylation of CpG islands, MeCP2 release, and MAGE-A11 gene re-activation, reinforcing the notion that the binding of MBPs with promoters depends on the methylation status of MAGE-A11 promoter. The reason why the binding of MBD1 on MAGE-A11 promoter could not be affected by DAC may be due to its unique structure and specific function in gene regulation. Beyond the conserved MBD domain at its N-terminal, it also has a transcriptional repression domain (TRD) and two or three specific CXXC domains distinct from other MBD-containing proteins. The first two CXXC domains (CXXC1 and CXXC2) allow MBD1 to bind to the methylated DNA, but the presence of the third CXXC domain (CXXC3) enables MBD1 to bind to DNA irrespective of its methylation status [48, 49]. MBD1 binds to methylated as well as unmethylated MAGE-A11 gene promoters, and leads to the repression of MAGE-A11 gene. Following MeCP2 binding to the methylated CpG sites, the recruitment of HDAC1 and HDAC2 was also increased, and the binding of H3Ac and H3K9Ac was decreased, leading to the deacetylation of histone and gene repression. This binding can be suppressed by demethylating agent DAC, suggesting that the subsequent histone acetylation after DNA methylation plays an accessory role in MAGE-A11 activation in ESCC progression.

In addition, MBPs functions as a link between DNA and histone methylation by forming complexes with histone methyltransferases to repress gene activation [50]. According to our present study, following MeCP2 binding to the methylated CpG sites, the occupancy of inactivation histone marks such as H3K27me3 and H3K9me3 was increased, whereas the occupancy of activation mark H3K4me3 was decreased. The occupancy of these marks can be regulated by demethylating agent DAC, suggesting that histone methylation is a subsequent event following MAGE-A11 promoter methylation. Another finding of our present study is that inhibiting the trimethylation of H3K27 by knockdown of EZH2 or DZNep increased MAGE-A11 expression in a DNA methylation-dependent manner.

Therefore, we sought to find the feasibility of modulating histone acetylation and methylation together with DNA methylation as a strategy to enhance MAGE-A11 activation under conditions potentially achievable in clinical settings [51–53]. Although DAC treatment could induce MAGE-A11 expression that might increase tumor growth, however, as a clinically used DNA methylation inhibitor, DAC treatment had clinical effects on tumors through promoting a lot of tumor suppressors that are commonly methylated in cancer progression [54, 55]. We recently reported epigenetic modulation by zebularine (another DNA methyltransferase inhibitor) - induced MAGE-A11 expression in breast cancer cells and facilitated cytotoxicity via MAGE-A11-specific cytotoxic T lymphocytes [56]. Our data support further development of TSA and DZNep, or other inhibitors of histone deacetylation and methylation for cancer immunotherapy targeting for MAGE-A11.

## MATERIALS AND METHODS

### Clinical specimens

All ESCC and the corresponding adjacent normal esophageal epithelial specimens were obtained from the patients who had ESCC and underwent the surgical treatment in our hospital from January 2009 to October 2009. All patients did not undergo the preoperative chemotherapy and radiotherapy. The clinicopathological parameters were collected from the case history. All patients were followed up for 12–60 months. This study was approved by the Medical Ethics Committee of our hospital.

### HE and immonuhistochemical (IHC) staining for tissue microarray (TMA)

TMAs construction, HE and IHC staining were performed and analyzed as described in our previous study [57].

### ESCC xenograft in mice

Three-week old nude mice were randomly divided into two groups (*n* = 3 per group). Human ESCC Eca109 cells were transfected with MAGE-A11 overexpression plasmid. The stable clones were subsequently established, and the transfection effects were examined with RT-PCR. Cells (5 × 10^6^) in 0.2 ml of PBS were harvested and injected subcutaneously into the flank region of nude mice. Mice were observed daily and palpated for tumor formation per two days. Two-dimensional tumor measurements were made after tumor formation. The tumor volume was calculated according to the formula: volume = π (short diameter^2^) × (long diameter)/6. After 22 days, the mice were sacrificed by cervical dislocation. Tumors were excised and used for IHC by using rabbit anti-human MAGE-A11 polyclonal antibody (Epitomics, California, USA), rabbit anti-human PCNA antibody (Proteintech, USA), and rabbit anti-human Ki76 antibody (Abcam, USA). The growth curve of the tumors was drawn.

### Cell culture and transfection

All cell lines were maintained in RPMI1640 (GIBCO, USA) supplemented with 10% heat-inactivated fetal bovine serum (GIBCO, USA), 50 units of penicillin and 50 μg/ml streptomycin. Cells were grown at 37°C in a water-saturated atmosphere of 5% CO_2_ in air. cDNA transfection and siRNA-mediated knockdown experiments were performed as described in our previous literature [58].

### RNA preparation, reverse transcription and quantitative real-time PCR (RT-qPCR)

Total RNA preparation and RT-qPCR were performed as described in our previous study [59]. PCR-based amplification was performed using the following primers in Supplementary Table 4.

### DNA preparation, sodium bisulfite treatment, methylation specific PCR (MSP), and Bisulfite sequencing PCR (BSP)

Genomic DNA was isolated by using a simplified Proteinase K (Merck, Darmstadt, Germany) digestion method. Bisulfite modification of DNA (1 µg) was performed by using an EZ DNA methylation-direct kit (Zymo Research, Irvine, CA, USA). The methylation status of MAGE-A11 promoter was detected by MSP. The primers for MSP were shown in Supplementary Table 5. MSP was performed by PCR using *GoTaq*^®^ *G2 Green* Master Mix (Promega, USA). The methylation status of each CpG site was confirmed by BSP. Bisulfite-modified DNA was subjected to PCR amplification. The PCR products were cloned into pGEM-T vectors (Promega, CA, USA) and 12 clones of each sample were selected for sequencing. The BSP primers were designed to recognize sodium bisulfite-converted DNA and encompassing CpG Island within the human MAGE-A11 (from −140 to +6 bp) (Supplementary Table 6).

### Western blot analysis

Cell lysates preparation and western blot were performed as described previously [59]. The primary antibodies for western blot were shown in Supplementary Table 7.

### Immunostaining

The immunostaining procedure was performed as described in our previous study [58].

### Chromatin immunoprecipitation assay (ChIP)

ChIP analysis was performed as described in our previous literature^50^. Immunoprecipitations were performed with 2 µg indicated antibodies or IgG. The primary antibodies for ChIP were shown in Supplementary Table S8. Primers used to measure the enrichment of MAGE-A11 promoter DNA sequence containing the CpG island are as follows: forward, 5′-CCC TCGCCACTACTTTTCC-3′; reverse, 5′-AGGAGCGAAC CCGATTCT-3′. The enrichment fold of ChIP DNA was calculated as percentage of input. The PCR products were resolved electrophoretically and visualized by ethidium bromide staining.

### Luciferase promoter assay

Cells were seeded at a density of 5 × 10^4^/12-well plate and cultured overnight. Cells were then transfected with the indicated MAGE-A11 promoter luciferase reporter plasmid, Renilla luciferase plasmid together with or without the expression plasmid for SP1, TFCP2 or ZEB1. Total amount of plasmid DNA was kept constant with the empty plasmid. Forty-eight hours after transfection, cell lysates were prepared and their luciferase activities were measured using dual luciferase reporter assay system (Promega, Madison, WI, USA).

### Cell proliferation assay

Cell proliferation assay was performed and analyzed as described in our previous study [59].

### Statistical analysis

All statistical analyses were performed with SPSS 22.0 software (SPSS Inc, Chicago, IL, USA). Results for RT-qPCR, ChIP, and luciferase reporter assay were analyzed by Student’s *t*-test. Chi-square test was used to analyze the association of MAGE-A11 expression with the cliniopathological parameters. Overall survival were estimated by the Kaplan-Meier method, differences between groups were compared were by the log-rank test. The Cox proportional hazards model was used for multivariate analysis to examine the potential prognostic value of different variables on overall survival. All statistical tests were two-sided, and *P* value less than 0.05 was considered statistically significant.

## SUPPLEMENTARY MATERIALS FIGURES AND TABLES



## References

[1] van der Bruggen P, Traversari C, Chomez P, Lurquin C, De Plaen E, Van den Eynde B, Knuth A, Boon T (1991). A gene encoding an antigen recognized by cytolytic T lymphocytes on a human melanoma. Science.

[2] Simpson AJ, Caballero OL, Jungbluth A, Chen YT, Old LJ (2005). Cancer/testis antigens, gametogenesis and cancer. Nat Rev Cancer.

[3] Sang M, Wang L, Ding C, Zhou X, Wang B, Wang L, Lian Y, Shan B (2011). Melanoma-associated antigen genes - an update. Cancer Lett.

[4] Sang M, Lian Y, Zhou X, Shan B (2011). MAGE-A family: attractive targets for cancer immunotherapy. Vaccine.

[5] Wang D, Wang J, Ding N, Li Y, Yang Y, Fang X, Zhao H (2016). MAGE-A1 promotes melanoma proliferation and migration through C-JUN activation. Biochem Biophys Res Commun.

[6] Liu W, Cheng S, Asa SL, Ezzat S (2008). The melanoma-associated antigen A3 mediates fibronectin-controlled cancer progression and metastasis. Cancer Res.

[7] Doyle JM, Gao J, Wang J, Yang M, Potts PR (2010). MAGE-RING protein complexes comprise a family of E3 ubiquitin ligases. Mol Cell.

[8] Feng Y, Gao J, Yang M (2011). When MAGE meets RING: insights into biological functions of MAGE proteins. Protein Cell.

[9] Pineda CT, Ramanathan S, Fon Tacer K, Weon JL, Potts MB, Ou YH, White MA, Potts PR (2015). Degradation of AMPK by a cancer-specific ubiquitin ligase. Cell.

[10] Pineda CT, Potts PR (2015). Oncogenic MAGEA-TRIM28 ubiquitin ligase downregulates autophagy by ubiquitinating and degrading AMPK in cancer. Autophagy.

[11] Shaw RJ (2015). Tumor Metabolism: MAGE-A Proteins Help TRIM Turn Over AMPK. Curr Biol.

[12] Monte M, Simonatto M, Peche LY, Bublik DR, Gobessi S, Pierotti MA, Rodolfo M, Schneider C (2006). MAGE-A tumor antigens target p53 transactivation function through histone deacetylase recruitment and confer resistance to chemotherapeutic agents. Proc Natl Acad Sci U S A.

[13] Yang B, O'Herrin SM, Wu J, Reagan-Shaw S, Ma Y, Bhat KM, Gravekamp C, Setaluri V, Peters N, Hoffmann FM, Peng H, Ivanov AV, Simpson AJ (2007). MAGE-A, mMage-b, and MAGE-C proteins form complexes with KAP1 and suppress p53-dependent apoptosis in MAGE-positive cell lines. Cancer Res.

[14] Wong PP, Yeoh CC, Ahmad AS, Chelala C, Gillett C, Speirs V, Jones JL, Hurst HC (2014). Identification of MAGEA antigens as causal players in the development of tamoxifen-resistant breast cancer. Oncogene.

[15] Laduron S, Deplus R, Zhou S, Kholmanskikh O, Godelaine D, De Smet C, Hayward SD, Fuks F, Boon T, De Plaen E (2004). MAGE-A1 interacts with adaptor SKIP and the deacetylase HDAC1 to repress transcription. Nucleic Acids Res.

[16] Bai S, He B, Wilson EM (2005). Melanoma antigen gene protein MAGE-11 regulates androgen receptor function by modulating the interdomain interaction. Mol Cell Biol.

[17] Liu Q, Su S, Blackwelder AJ, Minges JT, Wilson EM (2011). Gain in transcriptional activity by primate-specific coevolution of melanoma antigen-A11 and its interaction site in androgen receptor. J Biol Chem.

[18] Bai S, Grossman G, Yuan L, Lessey BA, French FS, Young SL, Wilson EM (2008). Hormone control and expression of androgen receptor coregulator MAGE-11 in human endometrium during the window of receptivity to embryo implantation. Mol Hum Reprod.

[19] Askew EB, Bai S, Blackwelder AJ, Wilson EM (2010). Transcriptional synergy between melanoma antigen gene protein-A11 (MAGE-11) and p300 in androgen receptor signaling. J Biol Chem.

[20] Su S, Blackwelder AJ, Grossman G, Minges JT, Yuan L, Young SL, Wilson EM (2012). Primate-specific melanoma antigen-A11 regulates isoform-specific human progesterone receptor-B transactivation. J Biol Chem.

[21] Lian Y, Sang M, Ding C, Zhou X, Fan X, Xu Y, Lu W, Shan B (2012). Expressions of MAGE-A10 and MAGE-A11 in breast cancers and their prognostic significance: a retrospective clinical study. J Cancer Res Clin Oncol.

[22] Sang M, Gu L, Liu F, Lian Y, Yin D, Fan X, Ding C, Huang W, Liu S, Shan B (2016). Prognostic Significance of MAGE-A11 in Esophageal Squamous Cell Carcinoma and Identification of Related Genes Based on DNA Microarray. Arch Med Res.

[23] James SR, Link PA, Karpf AR (2006). Epigenetic regulation of X-linked cancer/germline antigen genes by DNMT1 and DNMT3b. Oncogene.

[24] De Smet C, Lurquin C, Lethe B, Martelange V, Boon T (1999). DNA methylation is the primary silencing mechanism for a set of germ line- and tumor-specific genes with a CpG-rich promoter. Mol Cell Biol.

[25] Loriot A, De Plaen E, Boon T, De Smet C (2006). Transient down-regulation of DNMT1 methyltransferase leads to activation and stable hypomethylation of MAGE-A1 in melanoma cells. J Biol Chem.

[26] Rao M, Chinnasamy N, Hong JA, Zhang Y, Zhang M, Xi S, Liu F, Marquez VE, Morgan RA, Schrump DS (2011). Inhibition of histone lysine methylation enhances cancer-testis antigen expression in lung cancer cells: implications for adoptive immunotherapy of cancer. Cancer Res.

[27] Cedar H, Bergman Y (2009). Linking DNA methylation and histone modification: patterns and paradigms. Nat Rev Genet.

[28] Li LC, Dahiya R (2002). MethPrimer: designing primers for methylation PCRs. Bioinformatics.

[29] Takai D, Jones PA (2003). The CpG island searcher: a new WWW resource. In Silico Biol.

[30] Mathelier A, Fornes O, Arenillas DJ, Chen CY, Denay G, Lee J, Shi W, Shyr C, Tan G, Worsley-Hunt R, Zhang AW, Parcy F, Lenhard B (2016). JASPAR 2016: a major expansion and update of the open-access database of transcription factor binding profiles. Nucleic Acids Res.

[31] James SR, Cedeno CD, Sharma A, Zhang W, Mohler JL, Odunsi K, Wilson EM, Karpf AR (2013). DNA methylation and nucleosome occupancy regulate the cancer germline antigen gene MAGEA11. Epigenetics.

[32] Agathanggelou A, Honorio S, Macartney DP, Martinez A, Dallol A, Rader J, Fullwood P, Chauhan A, Walker R, Shaw JA, Hosoe S, Lerman MI, Minna JD (2001). Methylation associated inactivation of RASSF1A from region 3p21.3 in lung, breast and ovarian tumours. Oncogene.

[33] Ferguson AT, Evron E, Umbricht CB, Pandita TK, Chan TA, Hermeking H, Marks JR, Lambers AR, Futreal PA, Stampfer MR, Sukumar S (2000). High frequency of hypermethylation at the 14-3-3 sigma locus leads to gene silencing in breast cancer. Proc Natl Acad Sci U S A.

[34] Nan X, Ng HH, Johnson CA, Laherty CD, Turner BM, Eisenman RN, Bird A (1998). Transcriptional repression by the methyl-CpG-binding protein MeCP2 involves a histone deacetylase complex. Nature.

[35] Wischnewski F, Friese O, Pantel K, Schwarzenbach H (2007). Methyl-CpG binding domain proteins and their involvement in the regulation of the MAGE-A1, MAGE-A2, MAGE-A3, and MAGE-A12 gene promoters. Mol Cancer Res.

[36] Karpf AR, Bai S, James SR, Mohler JL, Wilson EM (2009). Increased expression of androgen receptor coregulator MAGE-11 in prostate cancer by DNA hypomethylation and cyclic AMP. Mol Cancer Res.

[37] Veljkovic J, Hansen U (2004). Lineage-specific and ubiquitous biological roles of the mammalian transcription factor LSF. Gene.

[38] Hansen U, Owens L, Saxena UH (2009). Transcription factors LSF and E2Fs: tandem cyclists driving G0 to S?. Cell Cycle.

[39] Santhekadur PK, Rajasekaran D, Siddiq A, Gredler R, Chen D, Schaus SE, Hansen U, Fisher PB, Sarkar D (2012). The transcription factor LSF: a novel oncogene for hepatocellular carcinoma. Am J Cancer Res.

[40] Yuedi D, Yuankun C, Jiaying Z, Han L, Yueqi W, Houbao L, Dexiang Z (2017). TFCP2 activates beta-catenin/TCF signaling in the progression of pancreatic cancer. Oncotarget.

[41] Jiang H, Du J, Jin J, Qi X, Pu Y, Fei B (2014). LSF expression and its prognostic implication in colorectal cancer. Int J Clin Exp Pathol.

[42] Postigo AA, Depp JL, Taylor JJ, Kroll KL (2003). Regulation of Smad signaling through a differential recruitment of coactivators and corepressors by ZEB proteins. EMBO J.

[43] Eger A, Aigner K, Sonderegger S, Dampier B, Oehler S, Schreiber M, Berx G, Cano A, Beug H, Foisner R (2005). DeltaEF1 is a transcriptional repressor of E-cadherin and regulates epithelial plasticity in breast cancer cells. Oncogene.

[44] Fontemaggi G, Gurtner A, Strano S, Higashi Y, Sacchi A, Piaggio G, Blandino G (2001). The transcriptional repressor ZEB regulates p73 expression at the crossroad between proliferation and differentiation. Mol Cell Biol.

[45] Postigo AA (2003). Opposing functions of ZEB proteins in the regulation of the TGFbeta/BMP signaling pathway. EMBO J.

[46] Sharma S, Kelly TK, Jones PA (2010). Epigenetics in cancer. Carcinogenesis.

[47] Wischnewski F, Pantel K, Schwarzenbach H (2006). Promoter demethylation and histone acetylation mediate gene expression of MAGE-A1, -A2, -A3, and -A12 in human cancer cells. Mol Cancer Res.

[48] Ng HH, Jeppesen P, Bird A (2000). Active repression of methylated genes by the chromosomal protein MBD1. Mol Cell Biol.

[49] Jorgensen HF, Ben-Porath I, Bird AP (2004). Mbd1 is recruited to both methylated and nonmethylated CpGs via distinct DNA binding domains. Mol Cell Biol.

[50] Hashimoto H, Vertino PM, Cheng X (2010). Molecular coupling of DNA methylation and histone methylation. Epigenomics.

[51] Steele N, Finn P, Brown R, Plumb JA (2009). Combined inhibition of DNA methylation and histone acetylation enhances gene re-expression and drug sensitivity *in vivo*. Br J Cancer.

[52] Moreno-Bost A, Szmania S, Stone K, Garg T, Hoerring A, Szymonifka J, Shaughnessy J, Barlogie B, Prentice HG, van Rhee F (2011). Epigenetic modulation of MAGE-A3 antigen expression in multiple myeloma following treatment with the demethylation agent 5-azacitidine and the histone deacetlyase inhibitor MGCD0103. Cytotherapy.

[53] Schrump DS, Fischette MR, Nguyen DM, Zhao M, Li X, Kunst TF, Hancox A, Hong JA, Chen GA, Pishchik V, Figg WD, Murgo AJ, Steinberg SM (2006). Phase I study of decitabine-mediated gene expression in patients with cancers involving the lungs, esophagus, or pleura. Clin Cancer Res.

[54] Nie J, Liu L, Li X, Han W (2014). Decitabine, a new star in epigenetic therapy: the clinical application and biological mechanism in solid tumors. Cancer Lett.

[55] Garcia-Manero G, Jabbour E, Borthakur G, Faderl S, Estrov Z, Yang H, Maddipoti S, Godley LA, Gabrail N, Berdeja JG, Nadeem A, Kassalow L, Kantarjian H (2013). Randomized open-label phase II study of decitabine in patients with low- or intermediate-risk myelodysplastic syndromes. J Clin Oncol.

[56] Zhang J, Sang M, Gu L, Liu F, Li W, Yin D, Wu Y, Liu S, Huang W, Shan B (2017). Zebularine Treatment Induces MAGE-A11 Expression and Improves CTL Cytotoxicity Using a Novel Identified HLA-A2-restricted MAGE-A11 Peptide. J Immunother.

[57] Sang M, Gu L, Yin D, Liu F, Lian Y, Zhang X, Liu S, Huang W, Wu Y, Shan B (2017). MAGE-A family expression is correlated with poor survival of patients with lung adenocarcinoma: a retrospective clinical study based on tissue microarray. J Clin Pathol.

[58] Sang M, Ando K, Okoshi R, Koida N, Li Y, Zhu Y, Shimozato O, Geng C, Shan B, Nakagawara A, Ozaki T (2009). Plk3 inhibits pro-apoptotic activity of p73 through physical interaction and phosphorylation. Genes Cells.

[59] Sang M, Hulsurkar M, Zhang X, Song H, Zheng D, Zhang Y, Li M, Xu J, Zhang S, Ittmann M, Li W (2016). GRK3 is a direct target of CREB activation and regulates neuroendocrine differentiation of prostate cancer cells. Oncotarget.

