# Supplementary material for: MAGE-A11 is activated through TFCP2/ZEB1 binding sites de-methylation as well as histone modification and facilitates ESCC tumor growth
Source: Oncotarget. 2017 Dec 5;9(3):3365–78. doi: 10.18632/oncotarget.22973 (PMC5790469; doi:10.18632/oncotarget.22973)
Supplement: Supplementary file 1 [file oncotarget-09-3365-s001.pdf]

MAGE-A11 is activated through TFCP2/ZEB1 binding sites de-methylation as well as histone modification and facilitates ESCC tumor growth

SUPPLEMENTARY MATERIALS

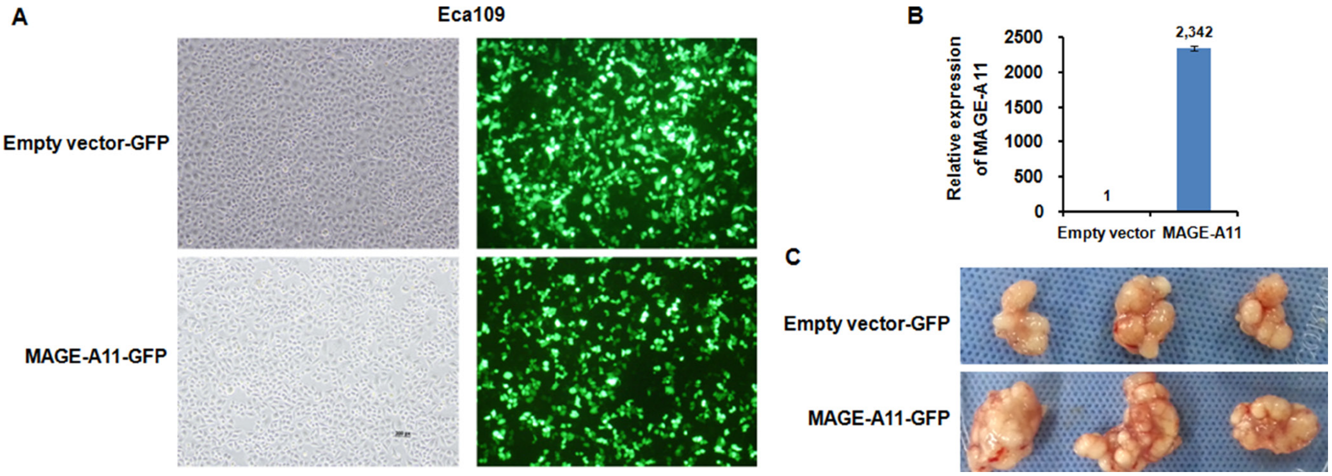

Supplementary Figure 1: (**A**) and (**B**) Transfection efficiency of MAGE-A11 in Eca109 cells. (**C**) Tumor size of Eca109 mice xenograft carrying MAGE-A11.

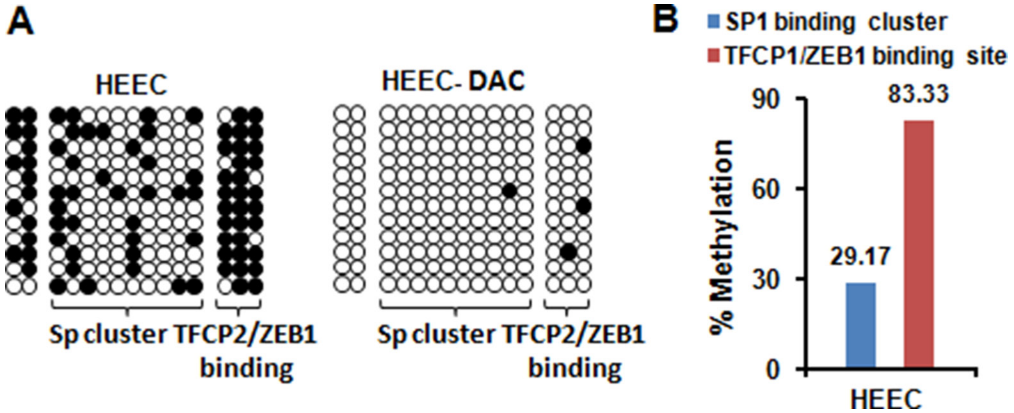

Supplementary Figure 2: The promoter of MAGE-A11 is hypermethylated in normal esophageal epithelial HEEC cells. (**A**) Bisulfite clonal sequencing showed the methylation status of the fifteen CG sites from –140 to +1 on MAGE-A11 promoter in HEEC cells before and after DAC treatment. (**B**) The methylation rate of the fifteen CG sites from –140 to +1 on MAGE-A11 promoter in HEEC cells.

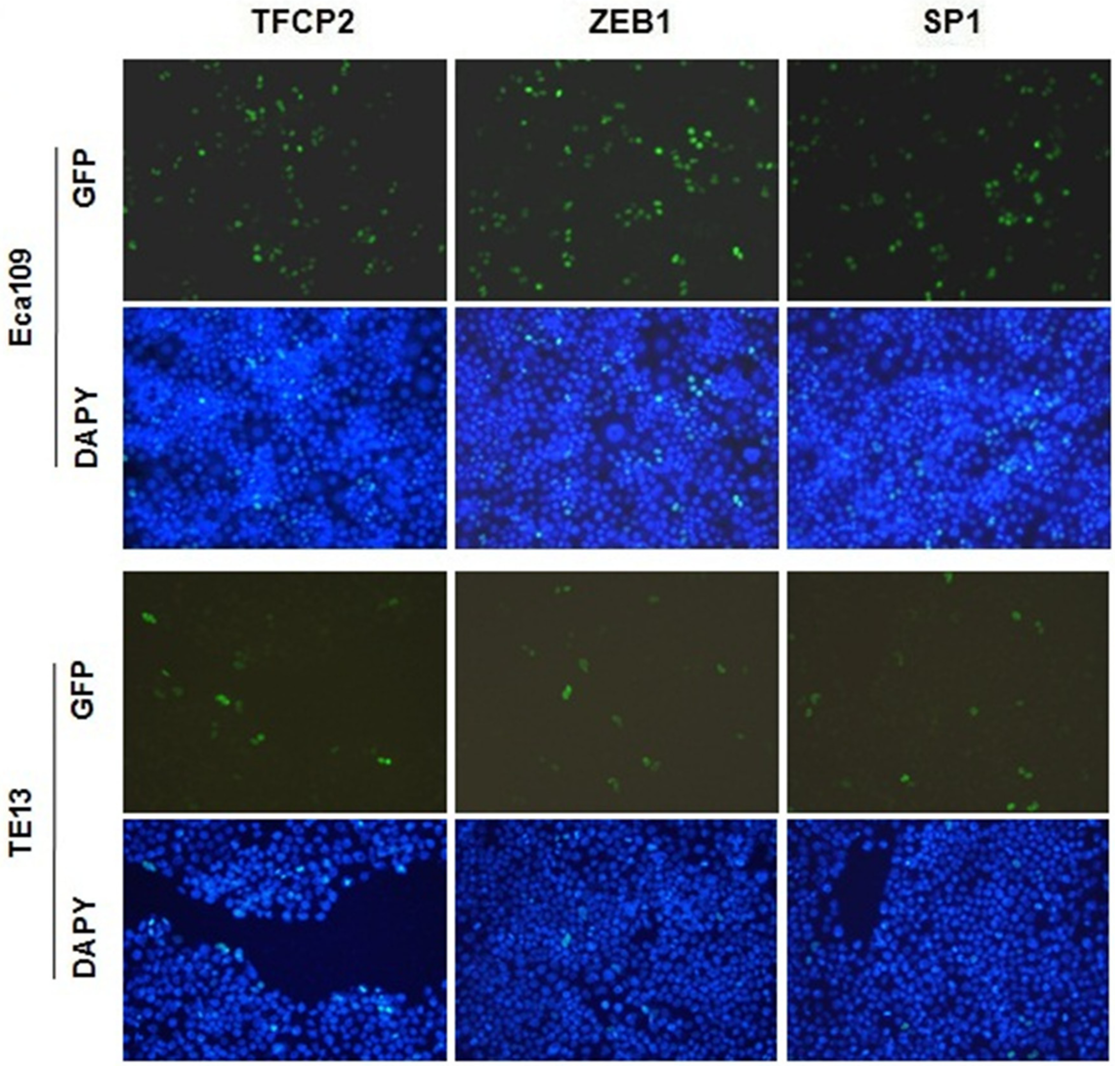

Supplementary Figure 3: The immunostaining experiment showed that TFCP2, ZEB1 and SP1 were detectable largely in cell nucleus.

Supplementary Table 1: Expression of MAGE-A11 in ESCC tissues and the corresponding adjacent normal esophageal epithelial tissues

Tissues	Cases	MAGE-A11		
		Negative	Positive	*P*
ESCC tissues	106	46	60	< 0.001
Normal esophageal epithelial tissues	106	106	0	

Supplementary Table 2: Correlation between MAGE-A11 expression and the clinicopathological parameters of ESCC patients

Variables	Cases	MAGE-A11		χ^2	*P*
		–	+		
**Age(y/s)**				0.485	0.486
**≤ 55**	29	11	18		
**> 55**	77	35	42		
**Gender**					
**Male**	72	30	42	0.273	0.601
**Female**	34	16	18		
**Tumor location**				0.189	0.664
**Cervical**	37	15	22		
**Thoracic**	69	31	38		
**WHO grade**				10.450	0.005
**G1**	10	4	6		
**G2**	63	35	28		
**G3–4**	33	7	26		
**Tumor invasion**				21.693	0.000
**T1–2**	58	37	21		
**T3–4**	48	9	39		
**Lymph node metastasis**				23.002	0.000
**N0**	18	17	1		
**N1**	88	29	59		
**TNM stage**				17.157	0.000
**I–II**	54	34	20		
**III–VI**	52	12	40		
**Distant metastasis or recurrence**				22.050	0.000
**M0**	68	41	27		
**M1**	38	5	33		

Supplementary Table 3: Cox multivariate analysis of prognostic factors on overall survival of ESCC patients

Variables	Univariate Analysis			Multivariate analysis		
	HR	95% CI	*p* Value	HR	95% CI	*p* Value
**Age**	1.200	0.722–1.994	0.482			
**Gender**	0.693	0.423–1.133	0.143			
**Tumor invasion**	4.546	3.259–6.343	< 0.001	1.411	0.798–2.496	0.236
**Lymph node metastasis**	9.084	5.376–15.438	< 0.001	2.604	1.245–5.446	0.011
**Distant metastasis or recurrence**	12.375	6.772–22.614	< 0.001	0.477	0.113-2.020	0.315
**TMN stage**	4.410	3.203–6.070	< 0.001	4.988	1.676–14.838	0.004
**Histological Grade**	1.753	1.150–2.673	0.009	1.130	0.760–1.681	0.546
**Tumor location**	0.843	0.518–1.372	0.492			
**MAGE-A11 expression**	4.694	2.711–8.128	< 0.001	4.457	2.331–8.524	< 0.001

Supplementary Table 4: Primers for RT-qPCR

Genes	Primers
MAGE-A11	Forward, 5′-CCTGACCTGATAGACCCTG-3′<br>Reverse, 5′-AGCATTTCTGCCTTTGTG-3′
SP1	Forward, 5′-CTGCCGCTCCCAACTTAC-3′<br>Reverse, 5′-TTGCCTCCACTTCCTCGA-3′
TFCP2	Forward, 5′-GAAACTCCATGATGAAACCCTA-3′<br>Reverse, 5′-TCCACCTCCAGCCCTCTA-3′
ZEB1	Forward, 5′-AAGTGGCGGTAGATGGTA-3′<br>Reverse, 5′-TTGTAGCGACTGGATTTT -3′
EZH2	Forward, 5′-CGCTTTTCTGTAGGCGATGT-3′<br>Reverse, 5′-TGGGTGTTGCATGAAAAGAA-3′
GAPDH	Forward, 5′-AGCCACATCGCTCAGACAC-3′<br>Reverse, 5′-GCCCAATACGACCAAATCC-3′

Supplementary Table 5: Primers for MSP

Genes	Primers
MAGE-A11 M	Forward, 5′-GTTTCGTTTTTATACGGGTAGAATC-3′<br>Reverse, 5′- TTCTCTCAAATCCCAAAAACG-3′
MAGE-A11 U	Forward, 5′-TGTTTTTATATGGGTAGAATTGG-3′<br>Reverse, 5′-TTCTCTCAAATCCCAAAAACAAA-3′

Supplementary Table 6: Primers for BSP

Genes	Primers
MAGE-A11	Forward, 5′-ATTTAGAGTTAGAGATTTGGT-3′<br>Reverse, 5′-ACTCCTCAATCCTCCCTCAAC-3′

Supplementary Table 7: Antibodies used for Western blot

Antibodies	Commercial Company
MAGE-A11	Abcam, USA
SP1	ACTIVE MOTIF, USA
TFCP2	BD Biosciences, USA
ZEB1	Santa Cruz Biotechnology, USA
H3	Proteintech, USA
Tubulin	Sino Biological Inc, China
MeCP2	Abcam, USA
EZH2	BD Transduction Laboratories, USA
H3K27me3	Cell Signaling, USA
β-actin	Abcam, USA

Supplementary Table 8: Antibodies used for ChIP

Antibodies	Commercial Company
SP1	ACTIVE MOTIF, USA
TFCP2	BD Biosciences, USA
ZEB1	Santa Cruz Biotechnology, USA
MBD1	Abcam, USA
HDAC1	Abcam, USA
HDAC2	Abcam, USA
H3K27me3	Cell Signaling, USA
H3K9me3	Cell Signaling, USA
H3K4me3	Cell Signaling, USA
